# Risk Assessment of Venous Thromboembolism in Neurocritical Patients: Construction and Validation of a Clinical Prediction Model

**DOI:** 10.1155/mi/8133560

**Published:** 2025-12-26

**Authors:** Meili Zhou, Rui Wang, Longhai Zhu, Chentao Wang, Weidong Hu, Jijun Shi

**Affiliations:** ^1^ Department of Neurology, The Second Affiliated Hospital of Soochow University, Suzhou, 215004, Jiangsu Province, China, suda.edu.cn; ^2^ Clinical Research Center of Neurological Disease, The Second Affiliated Hospital of Soochow University, Suzhou, 215004, Jiangsu Province, China, suda.edu.cn

**Keywords:** neurocritical care, neurologic intensive care unit, nomogram, prediction model, venous thromboembolism

## Abstract

**Background/Objective:**

Venous thromboembolism (VTE) remains a significant challenge in neurocritical care, with limited tailored risk assessment tools available for clinical practice. This study aimed to develop and validate a practical prediction model to support VTE prevention strategies in neurocritical patients.

**Methods:**

A total of 605 neurocritical patients were retrospectively enrolled in the neurologic intensive care unit (NICU) from May 2022 to April 2024. The eligible patients were randomly divided into a training dataset and a testing dataset in a ratio of 7:3. Variables with significant univariate effects in the training dataset were selected for multivariable stepwise regression analysis. The model fitting goodness was tested using the Hosmer–Lemeshow test, the area under the receiver operating characteristic (ROC) curve (AUC) was used to evaluate the model discrimination, and decision curve analysis (DCA) was used to test the clinical value of the model.

**Results:**

The final model identified age, length of stay in the NICU, activated partial thromboplastin time (APTT), D‐dimer, tracheotomy duration, pulmonary infection, antibiotic use, and dehydrating agents as key predictors. The nomogram demonstrated excellent discrimination (AUC 0.763, 95% CI: 0.714–0.811 in the training dataset; AUC 0.809, 95% CI: 0.732–0.885 in the testing cohort), strong calibration (Hosmer–Lemeshow test: *p* = 0.126 [training]; *p* = 0.823 [testing]) and consistent clinical net benefit across prevention thresholds (10%–40% risk probability).

**Conclusions:**

The risk prediction model developed in this study can effectively predict VTE occurrence in neurocritical patients with good discrimination and clinical utility, providing a valuable tool for identifying high‐risk individuals and performing early prevention and treatment measures.

## 1. Introduction

Venous thromboembolism (VTE), which includes deep vein thrombosis (DVT) and pulmonary embolism (PE), is a common complication in neurocritical patients [1]. Literature reports that the incidence of DVT after craniotomy in brain tumor patients is 10.5%−12.34% [2, 3], the VTE incidence in ischemic stroke patients is 2.5% [4], and the VTE incidence in patients with traumatic brain injury is 21% [5]. Neurocritical patients refer to those suffering from neurological diseases and have organ dysfunction (or are at risk of potential organ dysfunction). Due to paralysis and prolonged coma leading to blood stasis, their incidence of VTE is higher than other patients [6, 7].

In patients with severe neurological conditions, ~3/4 of thrombus occurrences are located in either one or both sided muscular veins. Literature reports that 50% of venous thrombi in the lower limbs originate from calf vein thrombi, and 50% of calf vein thrombi come from the intermuscular veins of the calf [8]. DVT incidence was 35.7% and 75.6% were in the muscular calf vein 1 week after neurologic intensive care unit (NICU) hospitalization [6]. DVT not only exacerbates patient suffering and diminishes quality of life but also increases the risk of in‐hospital mortality and medical expenses, thereby contributing to a substantial disease burden [9]. Consequently, the prevention and effective management of DVT are of paramount importance in clinical practice. Neurocritical care patients are presumed to be at high risk for VTE; however, data regarding risk factors in this population are limited [6, 10–13]. Therefore, it is crucial to explore the risk factors for VTE occurrence and establish a prediction model for neurocritical patients, so as to early identify, prevent, and treat neurocritical patients with high VTE risks.

Currently, clinical prediction models as quantitative tools for risk assessment are widely used in the health field. However, these models are less applied in VTE for neurocritical patients, and the quality and applicability of these models in clinical practice and future research remain unknown. A systematic review in 2021 evaluating risk assessment models for VTE in hospitalized adults demonstrated that Caprini RAM (22 studies), Padua prediction score (16 studies), IMPROVE models (8 studies), the Geneva risk score (4 studies), and the Kucher score (4 studies) have generally weak predictive accuracy [13]. There is insufficient evidence and too much heterogeneity to recommend the use of any particular RAM [13]. A 2024 meta‐analysis of nine DVT prediction models in acute stroke patients reported an incidence of 0.4%–28%, identified D‐dimer and age as common predictors, and showed area under the receiver operating characteristic (ROC) curves (AUCs) of 0.70–0.912, although all models carried a high risk of bias [14]. Meanwhile, the logistic regression analysis model can effectively explain the direction of variable effects and relative risk.

In this study, we develop an optimized VTE risk prediction model for neurocritical patients through integrated RF and logistic regression analyses, providing an evidence‐based tool for clinical prevention and management.

## 2. Materials and Methods

### 2.1. Participants

This study was designed as a single‐center retrospective cohort study. Patients with neurological critical illness who were admitted to the NICU of the Second Affiliated Hospital of Soochow University between May 2022 and April 2024 were enrolled in this study. Patients with hospitalization durations of 48 h or less or missing data were excluded. All participants received comprehensive VTE prophylaxis in accordance with current clinical guidelines [15, 16]. Enrolled patients were stratified into different VTE risk levels based on the Caprini score or Padua score, and prophylactic protocols were selected accordingly. Preventive measures included mechanical prophylaxis (intermittent pneumatic compression and lower‐limb elevation), pharmacologic prophylaxis with low‐molecular‐weight heparin when not contraindicated, or a combination of both. The selection and timing of prophylactic strategies were individually determined by experienced neurocritical care physicians according to each patient’s VTE risk profile and bleeding tendency, assessed using guideline‐recommended tools.

### 2.2. Data Collection

Demographic characteristics include age, gender, body mass index (BMI), risk factors, medical history, clinical laboratory parameters such as white blood cell (WBC) count, hemoglobin (Hb), platelet (PLT) count, albumin (ALB), total cholesterol (TC), triglycerides (TGs), high‐density lipoprotein cholesterol (HDL‐C), low‐density lipoprotein cholesterol (LDL‐C), homocysteine (HCY), D‐dimers, C‐reactive protein, etc., were collected. Comorbidities—including pulmonary infection, gastrointestinal bleeding, and epilepsy—were collected through the hospital information system during admission assessment and clinical management. Treatment modalities, such as thrombolytic therapy, anticoagulation, and anti‐PLT therapy, antibiotic use, and administration of dehydrating agents, as well as the duration of tracheotomy, length of stay in the ICU, and imaging findings, were also recorded.

### 2.3. Measurements of DVT and PE

In‐hospital VTE was defined as a newly diagnosed acute episode of DVT (involving either the upper or lower extremities) or PE, or both, confirmed by duplex venous ultrasonography, computed tomographic venography (CTV), computed tomographic pulmonary angiography (CTPA), or ventilation–perfusion (V/Q) lung scan, occurring more than 24 h after hospital admission [1, 10]. Lower extremity vascular ultrasound was performed using a Mindray portable color Doppler system (Model: M9) equipped with a high‐frequency linear probe (L12‐4S; 3–13 MHz). To monitor for DVT, routine duplex ultrasonography was conducted at the time of admission and was repeated during hospitalization if patients exhibited elevated D‐dimer levels, limb swelling, or localized pain. In cases where patients presented with elevated D‐dimer levels, tachypnea, abnormal electrocardiographic findings, signs of respiratory failure, or other clinical features highly suggestive of PE, CTPA was employed to confirm the diagnosis. However, due to the poor baseline condition and multiple comorbidities commonly observed in patients with severe neurological disorders, many were unable to tolerate this imaging procedure. In addition, multiple measures were implemented for the prevention of VTE. As a result, no cases of confirmed PE were identified in this study. All imaging assessments were conducted by a team of experienced radiologists. The occurrence of VTE and relevant clinical outcomes were documented throughout the hospital stay.

### 2.4. Statistical Analysis

Multiple imputation was used to handle missing values (missing rate <5%). The detailed procedures for data imputation and variable selection are provided in the Supporting Information. Categorical variables were presented as frequencies and percentages, and comparisons between groups were made using the *χ* ^2^ test or Fisher’s exact probability method; continuous variables that followed a normal distribution were statistically described as mean ± SD; if they did not follow a normal distribution, they were statistically described as M (P25, P75), and comparisons between groups were made using the *t*‐test or Mann–Whitney *U* test. The eligible patients were randomly divided into a training dataset (425 patients) and a testing dataset (180 patients) in a ratio of 7:3. Using whether to develop VTE as the dependent variable, univariate analysis was performed first. Multivariate Logistic regression analysis was performed by including all variables with *p*  < 0.05 in univariate analysis in the training dataset. The variance inflation factor (VIF) was used for collinearity diagnosis of independent variables, and VIF > 10 indicated multicollinearity between independent variables. The nomogram was established by the independent predictors for VTE and internally validated with the data of the training dataset, and externally validated with the data of the testing dataset. The model fitting goodness was tested using the Hosmer–Lemeshow test, the AUC was used to evaluate the model discrimination, and decision curve analysis (DCA) was used to test the clinical value of the model. Data analysis was performed using R4.3.2 software, and the nomogram was constructed based on the “rms” package. The significance level was set at two‐sided *α* = 0.05.

## 3. Results

### 3.1. Demographic and Clinical Characteristics of Neurocritical Patients in the Training Dataset and Testing Dataset

A total of 676 patients with neurological critical illness were enrolled retrospectively. In total, 605 patients were retrospectively analyzed after excluding 56 cases with missing data and 15 cases with less than 48 h of hospitalization. In the 605 neurocritical patients, the VTE incidence was 25.8% (156/605). The overall pattern of missing data was evaluated (Figure S1). The distribution of imputed data was highly consistent with the original data, supporting the reliability of the imputation results (Figure S2).

Baseline characteristics of the study population are summarized in Table 1, and detailed subgroup comparisons are provided in Table S1. The VIF results for all included variables are presented in Table S2. The results demonstrated that VTE patients were significantly older (training cohort: median 72.5 vs. 70.0 years, *p* = 0.046; testing cohort: 74.0 vs. 69.5 years, *p* = 0.009) and had substantially longer ICU stays (training: 16 vs. 10 days, *p*  < 0.001; testing: 17 vs. 9 days, *p*  < 0.001). Key laboratory differences included lower activated partial thromboplastin time (APTT) (training: 29.4 vs. 31.5 s, *p* = 0.025) and elevated D‐dimer levels (training: 2.14 vs. 1.52 μg/mL, *p* = 0.003; testing: 2.50 vs. 1.41 μg/mL, *p* = 0.006) in VTE patients. Clinical interventions showed significant associations, with higher rates of tracheostomy (training: 14.55% vs. 5.40%, *p* = 0.004; testing: 19.57% vs. 1.49%, *p*  < 0.001), antibiotic use (training: 94.55% vs. 78.10%, *p*  < 0.001), and dehydrating agent administration (training: 75.45% vs. 56.19%, *p*  < 0.001; testing: 80.43% vs. 52.24%, *p* = 0.001) in the VTE groups. No significant differences were observed in gender distribution, BMI, or most routine laboratory parameters between groups. These findings collectively identify critical risk factors that can inform surveillance protocols for high‐risk neurocritical patients.

**Table 1:** Comparison of demographic and clinical characteristics of patients with VTE and without VTE in the training dataset and testing dataset of neurocritical patients.

Variable	Training dataset (*n* = 425)			Testing dataset (*n* = 180)			
	Non‐VTE (*n* = 315, 74.12%)	VTE (*n* = 110, 25.88%)	*p*	Non‐VTE (*n* = 134, 74.44%)		VTE (*n* = 46, 25.56%)	*p*
Age (years)	70.00 (57.50–79.00)	72.50 (66.00–80.00)	0.046	69.50 (58.00–78.00)		74.00 (64.50–83.75)	0.009
Gender	—	—	0.661	—		—	0.683
Female, *n* (%)	111 (35.24%)	42 (38.18%)	—	46 (34.33%)		18 (39.13%)	—
Male, *n* (%)	204 (64.76%)	68 (61.82%)	—	88 (65.67%)		28 (60.87%)	—
BMI (kg/m^2^)	23.66 (21.25–26.60)	22.89 (20.99–24.99)	0.145	23.82 ± 4.04		23.96 ± 4.56	0.842
NLR	8.47 (5.06–13.08)	10.21 (6.40–15.42)	0.034	9.10 (4.94–14.67)		11.15 (4.83–17.02)	0.293
Creatinine (μmol/L)	66.00 (52.00–83.50)	72.50 (57.25–107.75)	0.042	67.00 (53.00–87.00)		74.50 (60.50–92.00)	0.188
CRP (mg/L)	10.30 (5.30–45.55)	17.55 (5.30–49.67)	0.570	10.85 (5.30–50.55)		11.00 (5.40–65.35)	0.618
PT (s)	13.30 (12.25–14.10)	13.36 (12.50–14.20)	0.408	13.30 (12.33–14.30)		13.50 (12.03–14.17)	0.651
APTT (s)	31.50 (26.30–36.05)	29.40 (24.88–34.75)	0.025	31.30 (25.97–37.38)		28.55 (26.27–34.20)	0.164
Fibrinogen (g/L)	3.31 (2.59–4.45)	3.70 (2.73–4.36)	0.179	3.41 (2.60–4.74)		3.48 (2.75–4.60)	0.836
D‐dimer (μg/mL)	1.52 (0.70–3.38)	2.14 (1.07–6.84)	0.003	1.41 (0.69–2.90)		2.50 (1.02–9.51)	0.006
INR	1.06 (1.00–1.14)	1.08 (1.03–1.17)	0.063	1.08 (1.00–1.15)		1.08 (1.00–1.18)	0.584
Length of ICU stay (day)	10.00 (5.00–16.00)	16.00 (11.00–28.00)	<0.001	9.00 (5.25–14.75)		17.00 (11.25–26.75)	<0.001
Length of CVC (day)	2.00 (0.00–12.00)	12.00 (1.00–22.00)	<0.001	0.00 (0.00–10.00)		14.50 (0.50–20.50)	<0.001
Length of tracheal intubation (day)	0.00 (0.00–2.00)	0.00 (0.00–10.00)	0.003	0.00 (0.00–4.00)		0.00 (0.00–10.00)	0.100
Length of ventilator use (day)	0.00 (0.00–2.00)	0.00 (0.00–12.25)	0.002	0.00 (0.00–4.00)		0.00 (0.00–15.00)	0.018
Length of tracheotomy (day)	0.00 (0.00–0.00)	0.00 (0.00–0.00)	0.002	0.00 (0.00–0.00)		0.00 (0.00–0.00)	<0.001
Etiology, *n* (%)	—	—	0.135	—		—	<0.001
Ischemic stroke	218 (69.21%)	80 (72.73%)	—	87 (64.93%)		33 (71.74%)	—
Intracerebral hemorrhage	14 (4.44%)	9 (8.18%)	—	5 (3.73%)		8 (17.39%)	—
Other	83 (26.35%)	21 (19.09%)	—	42 (31.34%)		5 (10.87%)	—
Complications, *n* (%)							
Pulmonary infection	135 (42.86%)	60 (54.55%)	0.045	47 (35.07%)		18 (39.13%)	0.752
Gastrointestinal bleeding	49 (15.56%)	37 (33.64%)	<0.001	25 (18.66%)		14 (30.43%)	0.143
Epilepsy	2 (0.63%)	0 (0.00%)	0.977	1 (0.75%)		1 (2.17%)	1.000
Electrolyte disturbance	189 (60.00%)	77 (70.00%)	0.080	68 (50.75%)		29 (63.04%)	0.203
Thrombolysis therapy, *n* (%)	25 (7.94%)	11 (10.00%)	0.638	12 (8.96%)		2 (4.35%)	0.492
CVC, *n* (%)	167 (53.02%)	84 (76.36%)	<0.001	66 (49.25%)		34 (73.91%)	0.006
Tracheal intubation (day)	109 (34.60%)	50 (45.45%)	0.056	49 (36.57%)		20 (43.48%)	0.512
Ventilator use (day)	108 (34.29%)	51 (46.36%)	0.032	49 (36.57%)		21 (45.65%)	0.360
Tracheotomy (day)	17 (5.40%)	16 (14.55%)	0.004	2 (1.49%)		9 (19.57%)	<0.001
Sedation, *n* (%)	115 (36.51%)	56 (50.91%)	0.011	56 (41.79%)		17 (36.96%)	0.688
Analgesia, *n* (%)	105 (33.33%)	49 (44.55%)	0.046	50 (37.31%)		21 (45.65%)	0.410
Vasoactive agent, *n* (%)	89 (28.25%)	33 (30.00%)	0.821	36 (26.87%)		14 (30.43%)	0.783
Antibiotic use, *n* (%)	246 (78.10%)	104 (94.55%)	<0.001	101 (75.37%)		40 (86.96%)	0.150
Dehydrating agent use, *n* (%)	177 (56.19%)	83 (75.45%)	<0.001	70 (52.24%)		37 (80.43%)	0.001

*Note: p*‐Values, AUCs, and ORs are presented to three decimal places; all other values are rounded to one decimal place. The same applies throughout.

Abbreviations: APTT, activated partial thromboplastin time; BMI, body mass index; CRP, C‐reactive protein; CVC, central venous catheterization; ICU, intensive care unit; INR, international normalized ratio; NLR, neutrophil to lymphocyte ratio; PT, prothrombin time; VTE, venous thromboembolism.

### 3.2. Multivariate Logistic Regression Analysis for the Risk Factors Associated With VTE

In the training set (Table 1), a single‐factor analysis was conducted on the variables included in the study between the two groups, and 21 variables had statistically significant differences between the two groups (*p* < 0.05). Table 2 presents the final multivariate logistic regression model for VTE risk prediction in neurocritical patients. The analysis identified eight significant predictors: age (OR = 1.013, 95% CI: 0.995–1.030, *p* = 0.158), length of ICU stay (OR = 1.056, 95% CI: 1.026–1.087, *p*  < 0.001), APTT (OR = 0.964, 95% CI: 0.936–0.992, *p* = 0.012), D‐dimer (OR = 1.035, 95% CI: 1.006–1.065, *p* = 0.018), length of tracheotomy (OR = 0.955, 95% CI: 0.923–0.987, *p* = 0.007), pulmonary infection (OR = 1.758, 95% CI: 0.996–3.105, *p* = 0.052), antibiotic use (OR = 2.246, 95% CI: 0.876–5.759, *p* = 0.092), and dehydrating agent use (OR = 1.610, 95% CI: 0.936–2.767, *p* = 0.085). Notably, prolonged ICU stay and elevated D‐dimer levels showed the strongest positive associations with VTE risk, while higher APTT values and longer tracheotomy duration appeared protective. The model demonstrated good predictive accuracy (AUC = 0.763 in training, 0.809 in testing) and calibration (Hosmer–Lemeshow *p* = 0.126), suggesting its potential utility for risk assessment in neurocritical care settings. These findings highlight modifiable clinical factors (e.g., infection control and medication management) that could be prioritized in nursing care plans for VTE prevention.

**Table 2:** Multivariate logistic regression analysis for the risk factors associated with VTE in neurocritical patients.

Variables	*β*	SE	*Z*	*p* Value	OR	95% CI
Constant	−2.927	0.840	−3.483	0.001	0.054	0.010–0.278
Age	0.012	0.009	1.413	0.158	1.013	0.995–1.030
Length of ICU stay	0.055	0.015	3.732	<0.001	1.056	1.026–1.087
APTT	−0.037	0.015	−2.516	0.012	0.964	0.936–0.992
D‐dimer	0.035	0.015	2.361	0.018	1.035	1.006–1.065
Length of tracheotomy day	−0.046	0.017	−2.711	0.007	0.955	0.923–0.987
Pulmonary infection	0.564	0.290	1.946	0.052	1.758	0.996–3.105
Antibiotic use	0.809	0.480	1.685	0.092	2.246	0.876–5.759
Dehydrating agent use	0.476	0.276	1.722	0.085	1.610	0.936–2.767

Abbreviations: APTT, activated partial thromboplastin time; ICU, intensive care unit.

### 3.3. Development of a Nomogram Predicting the Risk of VTE in Neurocritical Patients

**Figure 1:**
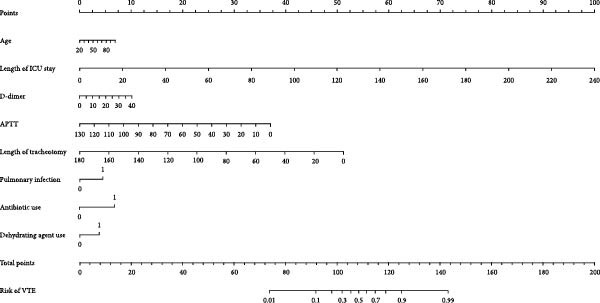
Nomogram for predicting the risk of VTE in neurocritical patients.

Figure 1 presents a clinically practical nomogram developed from the multivariate logistic regression model to predict individualized VTE risk in neurocritical patients. Based on the stepwise regression results, the VTE risk prediction model is established as follows: *Z* = −2.927 + 0.012 × age + 0.055 length of ICU stay − 0.037 × APTT + 0.035 × D‐dimer − 0.046 × length of tracheotomy + 0.564 × pulmonary infection + 0.809 × antibiotic use + 0.476 × dehydrating agent use.

Each variable was assigned weighted points on a 0–100 scale, with D‐dimer levels and ICU stay duration contributing most significantly to the total score. The clinicians can readily use this tool by plotting patient‐specific values for each parameter, summing the corresponding points, and mapping the total score to a predicted VTE probability ranging from <10% (low risk) to >60% (high risk). The nomogram’s alignment with established risk factors (e.g., D‐dimer) while incorporating neurocritical‐specific elements (e.g., dehydrating agents) enhances its clinical utility for clinical assessment. The clear graphical presentation facilitates rapid risk stratification without complex calculations, making it particularly valuable for time‐sensitive nursing interventions in critical care settings.

The predictors that ultimately entered the prediction model were age, length of ICU stay, APTT, D‐dimer, length of tracheotomy, pulmonary infection, antibiotic use, and dehydrating agent use.

### 3.4. The Evaluation and Validation of the Nomogram Model Predicting the Risk of VTE in Neurocritical Patients

Figures 2–4 collectively demonstrate the robust predictive performance and clinical utility of the VTE risk model. Figure 2 presents calibration plots showing excellent agreement between predicted and observed VTE probabilities in both training (mean absolute error = 0.034) and testing (mean absolute error = 0.032) cohorts, with Hosmer–Lemeshow *p*‐values of 0.126 and 0.823, respectively, indicating no significant deviation from perfect calibration. Figure 3 displays AUC values of 0.763 (95% CI: 0.714–0.811) for the training set and 0.809 (95% CI: 0.732–0.885) for the testing set, confirming the model’s strong discriminatory ability to distinguish high‐risk patients. Figure 4’s DCA reveals superior net benefit across clinically relevant probability thresholds (10%–40%) compared to “treat‐all” or “treat‐none” strategies, particularly in the 20%–30% risk range where preventive interventions would typically be considered. These results validate the model’s reliability for clinical decision‐making, as it consistently identifies true‐positive cases while minimizing unnecessary prophylaxis in low‐risk patients. The combination of statistical rigor and clinical interpretability makes this tool particularly valuable to implement risk‐stratified prevention protocols in neurocritical care units.

Figure 2The calibration chart of the nomogram model predicting VTE in the training dataset (A) and the testing dataset (B).

**Figure:**
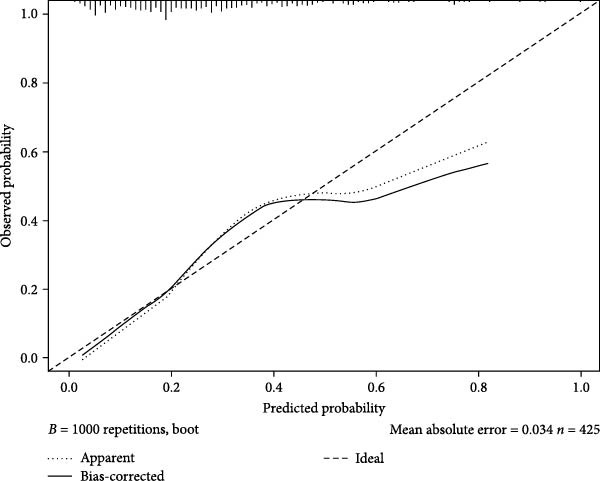
(A)

**Figure:**
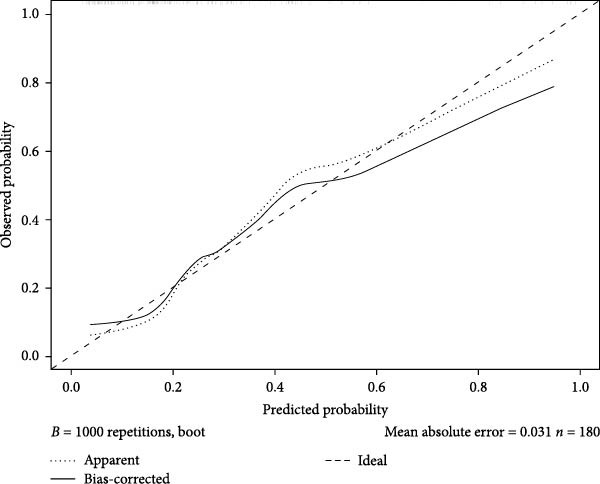
(B)

Figure 3Nomogram model predicts the ROC curve of VTE in the training dataset (A) and the testing dataset (B). ROC, receiver operating characteristic.

**Figure:**
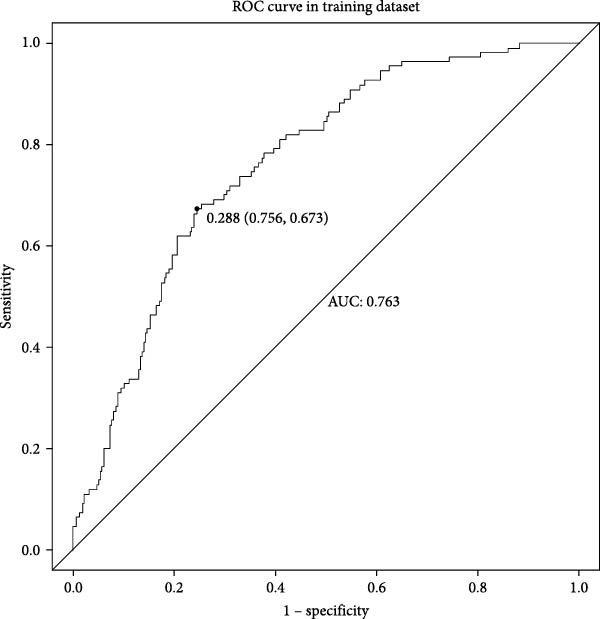
(A)

**Figure:**
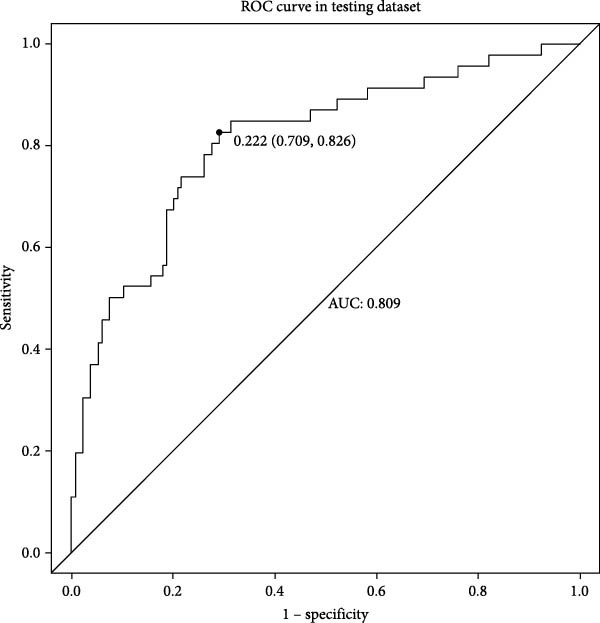
(B)

Figure 4The DCA of the nomogram model of VTE in the training dataset (A) and the testing dataset (B).

**Figure:**
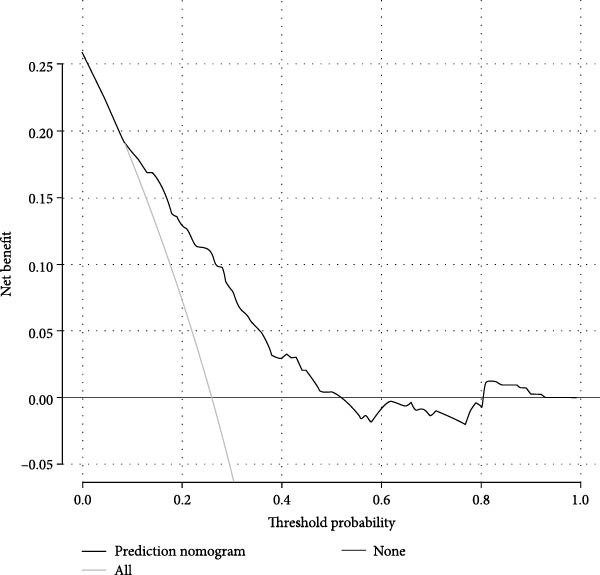
(A)

**Figure:**
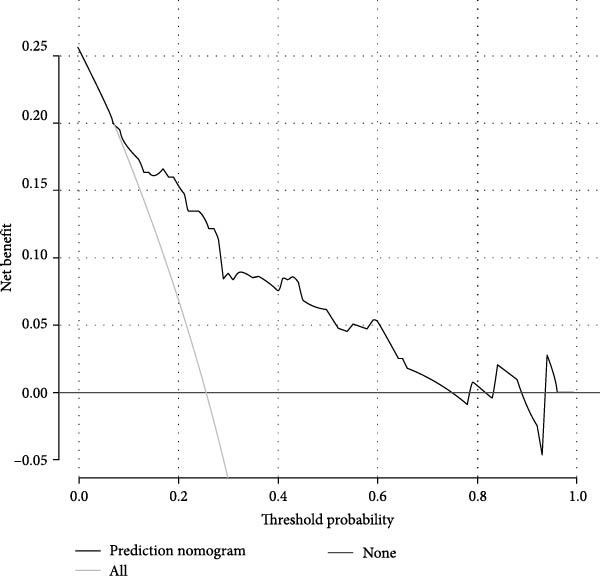
(B)

## 4. Discussion

This study successfully developed and validated a practical VTE risk prediction model specifically tailored for neurocritical care patients, addressing a critical gap in current assessment tools. Our findings demonstrate that eight clinically accessible variables—age, length of ICU stay, APTT, D‐dimer, tracheotomy duration, pulmonary infection, antibiotic use, and dehydrating agents—can effectively predict VTE risk. The model demonstrated excellent predictive accuracy with an AUC of 0.809 in the testing cohort, outperforming many existing general risk assessment tools. Compared with previous studies, this study integrates a more comprehensive set of laboratory parameters, clinical comorbidities, and invasive procedures, thereby enhancing its clinical relevance and applicability.

Due to paralysis and central nervous system disorders, patients in the neurocritical care unit are often at higher risk for VTE. All patients in our study underwent systematic VTE screening upon admission, followed by dynamic surveillance and re‐evaluation guided by clinical symptoms and coagulation parameters. This protocol significantly improved the detection rate of asymptomatic VTE. Although asymptomatic VTE may not pose an immediate life‐threatening risk in neurocritical patients, it can complicate therapeutic strategies and negatively impact long‐term outcomes.

This study developed a risk prediction model for VTE in neurocritical care patients by integrating an RF algorithm with logistic regression analysis, and visualized the model using a nomogram. The model demonstrated excellent discriminatory ability, achieving an AUC of 0.809, outperforming previously established models by Pan et al. (AUC = 0.756) [17] and Cheng et al. (AUC = 0.767) [18]. Calibration assessed via the Hosmer–Lemeshow goodness‐of‐fit test indicated satisfactory agreement between predicted and observed outcomes. Additionally, five‐fold cross‐validation confirmed the model’s stable predictive performance and robust discrimination, supporting its utility in the early identification of high‐risk patients and providing a practical reference for clinicians to implement timely preventive interventions.

Consistent with previous studies, this study identified age, length of hospital stay, and D‐dimer levels as significant factors associated with the development of VTE in neurocritical care patients [17, 19]. A variety of factors, including comorbidities such as stroke, diabetes, and frailty, age‐associated endothelial dysfunction, PLT functional alterations, and fluctuations in plasma components, collectively contribute to the elevated susceptibility to VTE observed in older adults [20]. Virchow’s triad of factors predisposing to thrombosis‐altered blood flow or stasis, changes in the composition of blood (hypercoagulability), and vessel wall damage, has been the foundation of the pathophysiology of VTE [21]. However, inflammatory mediators and immune cells are increasingly recognized as central players in thrombosis. Similarly, our study demonstrated that pulmonary infection and antibiotic administration are significant contributors to the development of VTE. Infection promotes thrombosis through complex, interrelated mechanisms. It triggers immune activation with upregulation of pro‐inflammatory cytokines, activates the coagulation cascade, suppresses fibrinolysis, and induces a hypercoagulable state [22]. Monocytes and neutrophils adhere to and interact with the venous endothelium, providing initiating stimuli for DVT [22]. Infection also induces exuberant formation of neutrophil extracellular traps, whose DNA–histone scaffold functions as a prothrombotic matrix that recruits PLTs and erythrocytes and facilitates fibrin polymerization, thereby promoting venous thrombus formation [23, 24]. In the ICU setting, concomitant factors—immobility, dehydration, fever, venous catheterization, and surgical or airway procedures—operationalize Virchow’s triad and accelerate the onset and progression of VTE [25]. Baseline analysis revealed that NLR levels were elevated in the VTE group compared to the non‐VTE group, further supporting this association. The Controlling Nutritional Status (CONUT) score, a widely recognized tool for assessing the risk of malnutrition, has also been shown to correlate with the degree of disability and the progression of infection in patients with stroke [26]. In our study, the CONUT score was included because most participants presented with severe stroke, making it a relevant marker of both nutritional status and prognosis in this neurocritical population. Although this variable was not retained in the final predictive model, its inclusion reflects the comprehensive and specialized nature of our study in the field of neurocritical care.

The superior predictive performance of the model developed in this study compared to existing models may be attributed to several factors. First, the use of an RF algorithm enabled effective selection of variables based on their relative importance, ensuring that only predictors with substantial contributions to the model were retained, thereby enhancing overall predictive accuracy. Second, the model incorporated both clinical characteristics and objective biochemical indicators, which improved the comprehensiveness and precision of VTE risk assessment. The model identifies several modifiable risk factors (e.g., pulmonary infection control, tracheotomy duration) that can be directly addressed through nursing interventions. Furthermore, the inclusion of key predictors such as D‐dimer levels and length of hospital stay—both recognized as significant risk factors for VTE—further strengthened the model’s discriminative capability. Therefore, the nomogram developed in this study can be utilized by healthcare professionals for dynamic risk stratification in neurocritical patients, facilitating the early identification of high‐risk individuals and enabling timely and effective preventive interventions.

This study has several limitations. First, as a retrospective study, it is subject to potential selection bias and unmeasured confounding factors. The absence of PE cases underscores the limitations of the current screening strategy and the diagnostic challenges in critically ill neurological patients, which may have led to missed asymptomatic or atypical cases and should be considered when interpreting the results. Second, due to limitations in data availability, important variables such as personal and family history of thrombosis, the Padua prediction score for medical patients, and the Caprini risk assessment model for surgical patients were not included. Third, external validation of the model has not yet been performed, and its generalizability remains to be further assessed. Although a nomogram was developed in this study, the evaluation of its clinical applicability was limited. We were unable to provide detailed operational guidelines, risk‐based management strategies, or electronic tools within the scope of this retrospective analysis. Despite alignment with contemporary VTE guidelines, real‐world heterogeneity in implementation and incomplete documentation preclude standardized quantification, potentially introducing residual confounding and affecting external validity. Future prospective studies will be needed to establish practical guidance, develop user‐friendly applications, and explore potential barriers and facilitators for clinical implementation based on existing literature.

Future work will focus on multicenter external validation. Specifically, we have initiated collaborations with tertiary hospitals and plan to recruit patients with neurological critical illness from different regions of Suzhou over the next 3 years, aiming to further assess the model’s generalizability in larger and more heterogeneous populations.

## 5. Conclusion

In conclusion, the VTE risk prediction model for neurocritical patients developed in this study has good predictive performance and clinical value. It can effectively predict the individualized risk of VTE occurrence in neurocritical patients, providing a reference for early prevention and treatment measures. Future research based on multicenter external validation, model optimization with prospective data, and integration with electronic health record systems is warranted to refine and recalibrate the model, thereby enhancing its applicability in real‐world clinical settings.

## Ethics Statement

The study protocol was approved by the Ethics Committee of the Second Affiliated Hospital of Soochow University (JD‐HG‐2025‐059) and performed in accordance with the ethical standards laid down in the 1964 Declaration of Helsinki and its later amendments. Written informed consent was obtained from all participants or their legal guardians.

## Consent

The authors have nothing to report.

## Disclosure

All authors approved the protocol.

## Conflicts of Interest

The authors declare no conflicts of interest.

## Author Contributions

**Jijun Shi and Weidong Hu:** study concept and design. **Meili Zhou and Rui Wang:** thesis writing and revision. **Meili Zhou and Chentao Wang:** acquisition of data. **Longhai Zhu:** statistical analysis and interpreted the data. **Jijun Shi and Weidong Hu:** study supervision. Meili Zhou, Rui Wang, and Longhai Zhu contributed equally to this work and should be considered co‐first authors. Jijun Shi and Weidong Hu contributed equally.

## Funding

This work was supported by the Shaanxi Provincial Natural Science Basic Research Program (2025JC‐YBMS‐1020), Nuclear Medical Technology Innovation Key Project (ZHYLZD2025018), Suzhou Science and Technology Development Plan Project (SKY2023180), Clinical Research Center of Neurological Disease of the Second Affiliated Hospital of Soochow University (ND2023B06), Jiangsu Provincial Medical Key Discipline (ZDXK202217), and Project of China International Medical Foundation (2022‐N‐01‐26).

## Supporting Information

Additional supporting information can be found online in the Supporting Information section.

## Supporting information


**Supporting Information** Figure S1: Visualization of missing data patterns. Figure S2: Distribution of imputed versus original data. Table S1: Comparison of demographic and clinical characteristics of patients with VTE and without VTE in the training dataset and testing dataset of neurocritical patients. Table S2: VIF values for the included variables.

## Data Availability

The data that support the findings of this study are available from the corresponding author upon reasonable request.

## References

[1] Khan F. , Tritschler T. , Kahn S. R. , and Rodger M. A. , Venous Thromboembolism, The Lancet. (2021) 398, no. 10294, 64–77, 10.1016/S0140-6736(20)32658-1. 33984268

[2] Guo X. , Zhang F. , and Wu Y. , et al.Coagulation Alteration and Deep Vein Thrombosis in Brain Tumor Patients During the Perioperative Period, World Neurosurgery. (2018) 114, e982–e991, 10.1016/j.wneu.2018.03.128, 2-s2.0-85045324314. 29588239

[3] Shi S. , Cheng J. , Chen H. , Zhang Y. , Zhao Y. , and Wang B. , Preoperative and Intraoperative Predictors of Deep Venous Thrombosis in Adult Patients Undergoing Craniotomy for Brain Tumors: A Chinese Single-Center, Retrospective Study, Thrombosis Research. (2020) 196, 245–250, 10.1016/j.thromres.2020.09.005. 32919179

[4] Indredavik B. , Rohweder G. , Naalsund E. , and Lydersen S. , Medical Complications in a Comprehensive Stroke Unit and an Early Supported Discharge Service, Stroke. (2008) 39, no. 2, 414–420, 10.1161/STROKEAHA.107.489294, 2-s2.0-39349088303. 18096834

[5] Gearhart M. M. , Luchette F. A. , and Proctor M. C. , et al.The Risk Assessment Profile Score Identifies Trauma Patients at Risk for Deep Vein Thrombosis, Surgery. (2000) 128, no. 4, 631–640, 10.1067/msy.2000.108224, 2-s2.0-0033772010. 11015097

[6] Zhang P. , Bian Y. , and Xu F. , et al.The Incidence and Characteristics of Venous Thromboembolism in Neurocritical Care Patients: A Prospective Observational Study, Clinical and Applied Thrombosis/Hemostasis. (2020) 26, 10.1177/1076029620907954, 1076029620907954. 32090609 PMC7288821

[7] Nyquist P. , Bautista C. , and Jichici D. , et al.Prophylaxis of Venous Thrombosis in Neurocritical Care Patients: An Evidence-Based Guideline: A Statement for Healthcare Professionals From the Neurocritical Care Society, Neurocritical Care. (2016) 24, no. 1, 47–60, 10.1007/s12028-015-0221-y, 2-s2.0-84957437926. 26646118

[8] Galanaud J.-P. , Sevestre M.-A. , and Genty C. , et al.Comparison of the Clinical History of Symptomatic Isolated Muscular Calf Vein Thrombosis Versus Deep Calf Vein Thrombosis, Journal of Vascular Surgery. (2010) 52, no. 4, 932–938.e2, 10.1016/j.jvs.2010.05.019, 2-s2.0-77957601694. 20630688

[9] Xie W. , Lu T. , and Yang X. , et al.Prevention of Deep Vein Thrombosis in Patients With Aneurysmal Subarachnoid Hemorrhage: A Best Practice Implementation Project, BMC Nursing. (2024) 23, no. 1, 10.1186/s12912-024-02463-1, 793. 39472950 PMC11523577

[10] Viarasilpa T. , Panyavachiraporn N. , and Jordan J. , et al.Venous Thromboembolism in Neurocritical Care Patients, Journal of Intensive Care Medicine. (2020) 35, no. 11, 1226–1234, 10.1177/0885066619841547, 2-s2.0-85065513368. 31060441

[11] Samuel S. , Iluonakhamhe E. K. , and Adair E. , et al.High Dose Subcutaneous Unfractionated Heparin for Prevention of Venous Thromboembolism in Overweight Neurocritical Care Patients, Journal of Thrombosis and Thrombolysis. (2015) 40, no. 3, 302–307, 10.1007/s11239-015-1202-x, 2-s2.0-84940435781. 25736986

[12] Alshaya A. I. , Alyahya H. , and Alzoman R. , et al.Chemical Versus Mechanical and Chemical Venous Thromboembolism Prophylaxis in Neurocritically Ill Patients: A Cohort Study, Clinical Pharmacology: Advances and Applications. (2023) 15, 1–8, 10.2147/CPAA.S388950. 36644519 PMC9833649

[13] Pandor A. , Tonkins M. , and Goodacre S. , et al.Risk Assessment Models for Venous Thromboembolism in Hospitalised Adult Patients: A Systematic Review, BMJ Open. (2021) 11, no. 7, 10.1136/bmjopen-2020-045672, e045672. PMC8323381 34326045

[14] Fu H. , Hou D. , and Xu R. , et al.Risk Prediction Models for Deep Venous Thrombosis in Patients With Acute Stroke: A Systematic Review and Meta-Analysis, International Journal of Nursing Studies. (2024) 149, 10.1016/j.ijnurstu.2023.104623, 104623. 37944356

[15] Kearon C. , Akl E. A. , and Comerota A. J. , et al.Antithrombotic Therapy for VTE Disease: Antithrombotic Therapy and Prevention of Thrombosis, 9th ed: American College of Chest Physicians Evidence-Based Clinical Practice Guidelines, Chest. (2012) 142, no. 6, e419S–e496S. 10.1378/chest.11-2301 PMC3278049 22315268

[16] Witt D. M. , Nieuwlaat R. , and Clark N. P. , et al.American Society of Hematology 2018 Guidelines for Management of Venous Thromboembolism: Optimal Management of Anticoagulation Therapy, Blood Advances. (2018) 2, no. 22, 3257–3291, 10.1182/bloodadvances.2018024893, 2-s2.0-85057278883. 30482765 PMC6258922

[17] Pan X. , Wang Z. , Chen Q. , Xu L. , and Fang Q. , Development and Validation of a Nomogram for Lower Extremity Deep Venous Thrombosis in Patients After Acute Stroke, Journal of Stroke and Cerebrovascular Diseases. (2021) 30, no. 5, 10.1016/j.jstrokecerebrovasdis.2021.105683, 105683. 33676327

[18] Cheng H.-R. , Huang G.-Q. , and Wu Z.-Q. , et al.Individualized Predictions of Early Isolated Distal Deep Vein Thrombosis in Patients With Acute Ischemic Stroke: A Retrospective Study, BMC Geriatrics. (2021) 21, no. 1, 10.1186/s12877-021-02088-y, 140. 33632136 PMC7908755

[19] Wang Y. , Shi Y. , Dong Y. , Dong Q. , Ye T. , and Fang K. , Clinical Risk Factors of Asymptomatic Deep Venous Thrombosis in Patients With Acute Stroke, Clinical and Applied Thrombosis/Hemostasis. (2019) 25, 10.1177/1076029619868534, 2-s2.0-85071522997, 1076029619868534. 31434499 PMC6829645

[20] Tritschler T. and Aujesky D. , Venous Thromboembolism in the Elderly: A Narrative Review, Thrombosis Research. (2017) 155, 140–147, 10.1016/j.thromres.2017.05.015, 2-s2.0-85019560921. 28550759

[21] Colling M. E. , Tourdot B. E. , and Kanthi Y. , Inflammation, Infection and Venous Thromboembolism, Circulation Research. (2021) 128, no. 12, 2017–2036, 10.1161/CIRCRESAHA.121.318225. 34110909 PMC8202069

[22] Gaertner F. and Massberg S. , Blood Coagulation in Immunothrombosis—At the Frontline of Intravascular Immunity, Seminars in Immunology. (2016) 28, no. 6, 561–569, 10.1016/j.smim.2016.10.010, 2-s2.0-85003680946. 27866916

[23] Fuchs T. A. , Brill A. , and Duerschmied D. , et al.Extracellular DNA Traps Promote Thrombosis, Proceedings of the National Academy of Sciences. (2010) 107, no. 36, 15880–15885, 10.1073/pnas.1005743107, 2-s2.0-77957652949. PMC2936604 20798043

[24] Brill A. , Fuchs T. A. , and Savchenko A. S. , et al.Neutrophil Extracellular Traps Promote Deep Vein Thrombosis in Mice, Journal of Thrombosis and Haemostasis. (2012) 10, no. 1, 136–144, 10.1111/j.1538-7836.2011.04544.x, 2-s2.0-84855414173. 22044575 PMC3319651

[25] Schmidt M. , Horvath-Puho E. , Thomsen R. W. , Smeeth L. , and Sørensen H. T. , Acute Infections and Venous Thromboembolism, Journal of Internal Medicine. (2012) 271, no. 6, 608–618, 10.1111/j.1365-2796.2011.02473.x, 2-s2.0-84861230280. 22026462 PMC3505369

[26] Di Vincenzo O. , D.’Elia L. , Ballarin G. , Pasanisi F. , and Scalfi L. , Controlling Nutritional Status (CONUT) Score and the Risk of Mortality or Impaired Physical Function in Stroke Patients: A Systematic Review and Meta-Analysis, Nutrition, Metabolism and Cardiovascular Diseases. (2023) 33, no. 8, 1501–1510, 10.1016/j.numecd.2023.05.012. 37336716

