# Supplementary material for: Risk Assessment of Venous Thromboembolism in Neurocritical Patients: Construction and Validation of a Clinical Prediction Model
Source: Mediators Inflamm. 2025 Dec 26;2025:8133560. doi: 10.1155/mi/8133560 (PMC12767376; doi:10.1155/mi/8133560)
Supplement: Supplementary file 1 — Supporting Information Figure S1: Visualization of missing data patterns. Figure S2: Distribution of imputed versus original data. Table S1: Comparison of demographic and clinical characteristics of patients with VTE and without VTE in the training dataset and testing dataset of neurocritical patients. Table S2: VIF values for the included variables. [file MI-2025-8133560-s001.docx]

**Supplementary Materials**

**Methods:**

A total of 605 patients were included after data curation,. Four variables with a missing rate >5% (LDL, HDL, glycated hemoglobin, and plasma homocysteine) were excluded as they were not suitable for imputation. Multiple imputation was performed using the default predictive mean matching method with 5 imputations. Seven variables were imputed: D-dimer, PT, APTT, Fib, INR, TG, TC, and FAR (fibrinogen-to-albumin ratio). Since FAR was a derived variable, it was removed prior to imputation and recalculated after imputation to ensure reliability. Based on the imputation performance, the second imputed dataset was selected as the final output. Ultimately, a dataset named vte_new was obtained, consisting of 605 patients and 71 variables.

**Results:**

**Supplementary Table 1 Comparison of demographic and clinical characteristics of patients with VTE and without** **VTE** **in the training dataset and testing dataset of** **neurocritical patients**

| **Variables** | **Training dataset（n=425）** | | | | **Testing dataset（n=180）** | | | |
| --- | --- | --- | --- | --- | --- | --- | --- | --- |
|  | **non-VTE**  **（n=315, 74.12%）** | **VTE**  **（n=110, 25.88%）** | ***P*** | **Non-VTE**  **（n=134, 74.44%）** | | **VTE**  **（n=46, 25.56%）** | ***P*** |  |
| white cell, 10^9^/L | 10.00 (8.10-13.10) | 9.70 (8.00-12.40) | 0.637 | 9.60 (7.90-12.12) | | 10.35 (7.95-13.55) | 0.215 |  |
| Neutrophil, 10^9^/L | 8.40 (6.30-11.20) | 8.35 (6.40-10.70) | 0.963 | 8.20 (6.10-10.50) | | 8.80 (6.25-11.88) | 0.190 |  |
| Lymphocyte, 10^9^/L | 1.00 (0.70-1.40) | 0.90 (0.60-1.20) | **0.012** | 0.90 (0.60-1.30) | | 1.00 (0.60-1.30) | 0.955 |  |
| Albumin, g/L | 38.50 (34.60-41.75) | 38.15 (34.20-40.77) | 0.200 | 38.75 (34.82-42.00) | | 38.75 (35.00-42.05) | 0.769 |  |
| Triglycerides, mmol/L | 1.09 (0.80-1.56) | 1.09 (0.79-1.51) | 0.533 | 1.15 (0.82-1.59) | | 1.00 (0.76-1.30) | 0.200 |  |
| Total cholesterol, mmol/L | 4.06 (3.26-5.00) | 3.87 (3.22-4.65) | 0.295 | 4.18±1.23 | | 4.30±1.27 | 0.574 |  |
| Count score | 3.00 (2.00-5.00) | 4.00 (3.00-5.00) | **0.026** | 3.00 (2.00-5.00) | | 3.00 (2.00-5.00) | 0.834 |  |
| Risk factors, n (%) |  |  |  |  | |  |  |  |
| Smoking history | 107 (33.97%) | 32 (29.09%) | 0.412 | 45 (33.58%) | | 16 (34.78%) | 1.000 |  |
| Drinking history | 47 (14.92%) | 22 (20.00%) | 0.274 | 17 (12.69%) | | 7 (15.22%) | 0.854 |  |
| Hypertension | 203 (64.44%) | 80 (72.73%) | 0.142 | 85 (63.43%) | | 39 (84.78%) | **0.012** |  |
| Diabetes mellitus | 82 (26.03%) | 33 (30.00%) | 0.495 | 35 (26.12%) | | 17 (36.96%) | 0.226 |  |
| Atrial fibrillation | 53 (16.83%) | 24 (21.82%) | 0.305 | 15 (11.19%) | | 7 (15.22%) | 0.647 |  |
| Previous stroke | 77 (24.44%) | 29 (26.36%) | 0.785 | 39 (29.10%) | | 11 (23.91%) | 0.626 |  |
| Coronary heart disease | 21 (6.67%) | 9 (8.18%) | 0.751 | 9 (6.72%) | | 3 (6.52%) | 1.000 |  |
| Hyperlipidemia | 6 (1.90%) | 2 (1.82%) | 1 | 4 (2.99%) | | 2 (4.35%) | 1 |  |
| Medication history, n (%) |  |  | 0.415 |  | |  | 0.764 |  |
| Anticoagulant therapy | 25 (7.94%) | 13 (11.82%) |  | 10 (7.46%) | | 2 (4.35%) |  |  |
| Antiplatelet therapy | 45 (14.29%) | 13 (11.82%) |  | 26 (19.40%) | | 9 (19.57%) |  |  |

*P*-values, AUCs, and ORs are presented to three decimal places; all other values are rounded to one decimal place. Same applies throughout.

Abbreviations: VTE, venous thromboembolism; BMI, body mass index; ICU, intensive care unit; NLR, neutrophil to lymphocyte ratio; CRP, C-reactive protein; PT, prothrombin time; APTT, activated partial thromboplastin time; INR, international normalized ratio; CVC, central venous catheterization **;** CONUT score: Controlling Nutritional Status score


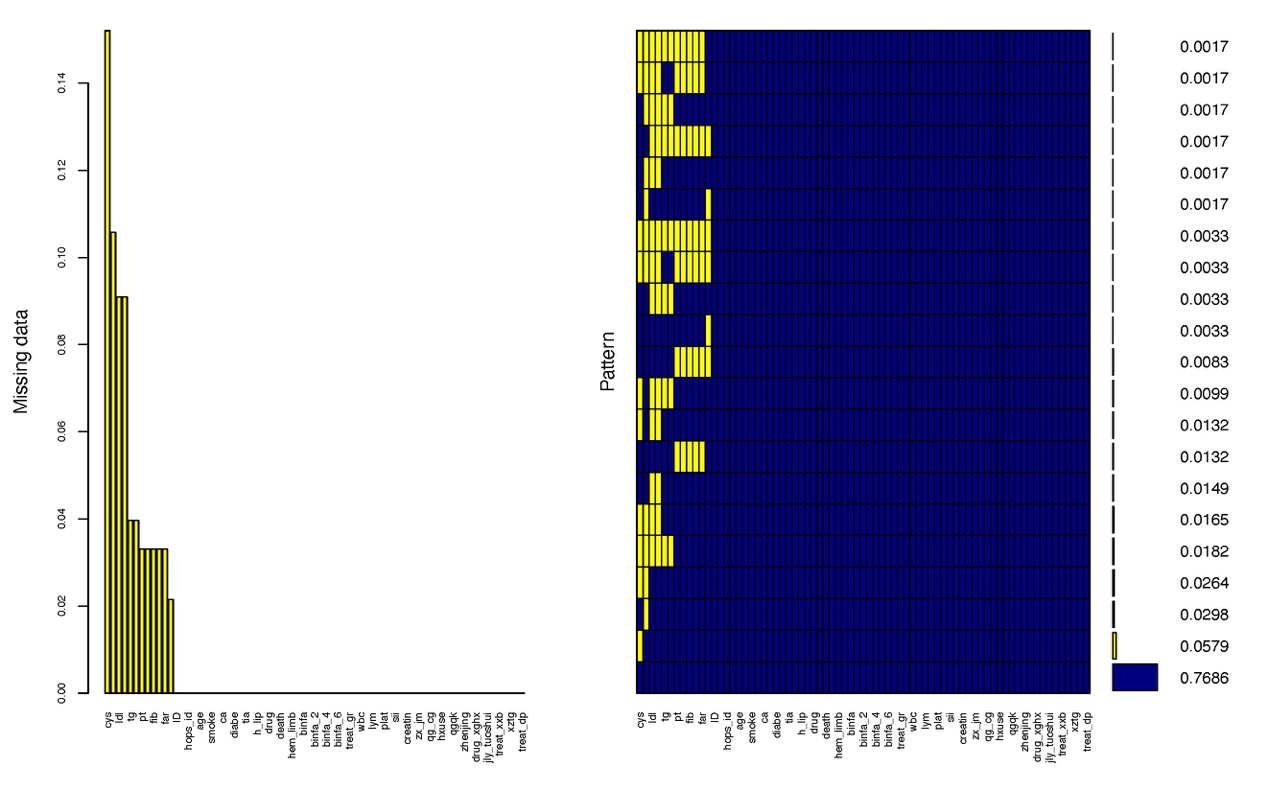


**Figure S1. Visualization of missing data patterns**

The bar plot (left) shows the proportion of missing values for each variable, with most variables below 5% and a few approaching 15%. The heatmap (right) illustrates the missing data patterns, where blue represents observed and yellow represents missing values. Approximately 76.9% of cases contained complete data without any missing values.


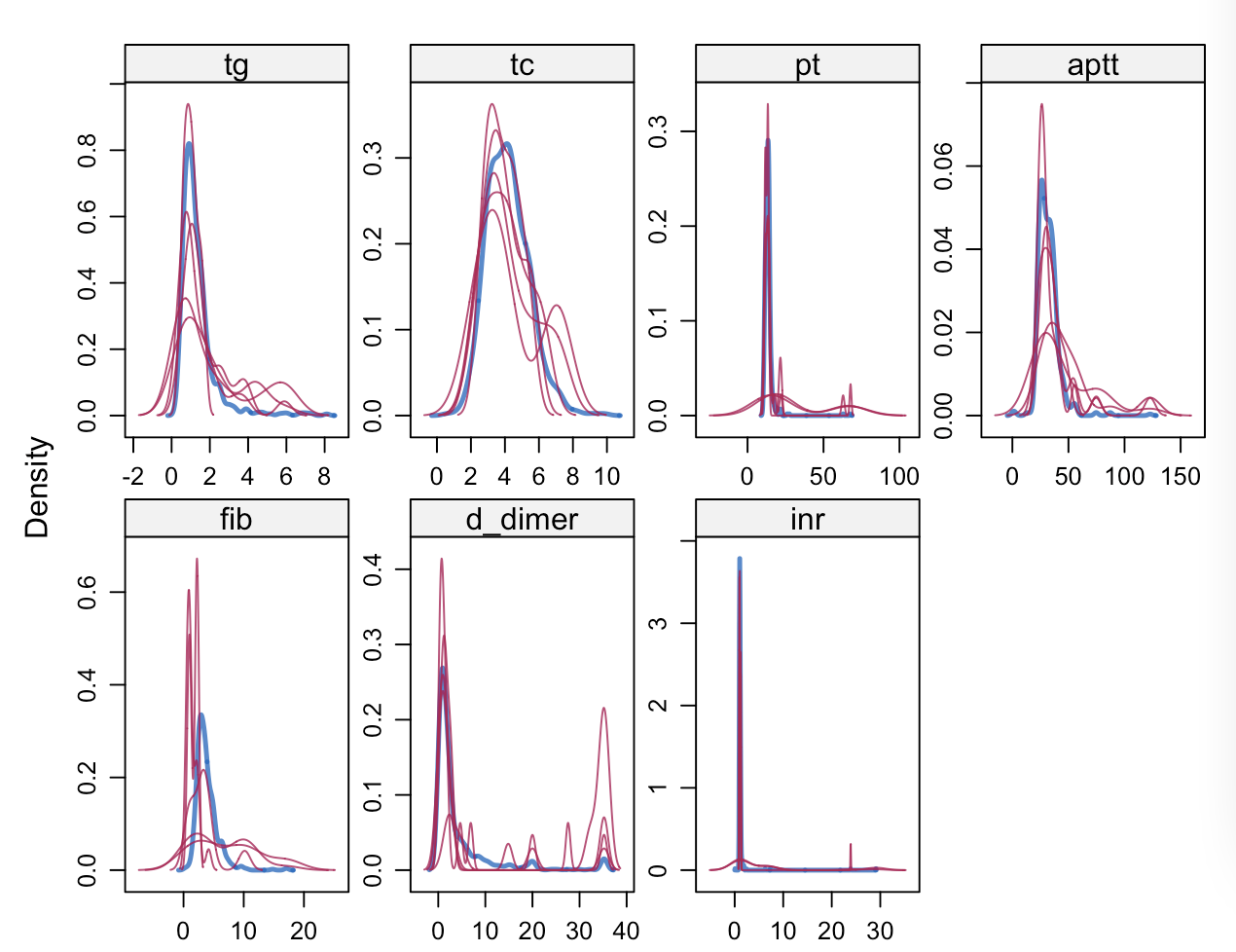


**Figure S2. Distribution of imputed versus original data**

Density plots of seven laboratory variables, including triglyceride (TG), total cholesterol (TC), prothrombin time (PT), activated partial thromboplastin time (APTT), fibrinogen (Fib), D-dimer, and international normalized ratio (INR), are shown. The blue curves represent the distribution of the original data, while the red curves represent the distributions of the imputed datasets. The imputed data closely overlapped with the original data, indicating that the multiple imputation procedure preserved the overall data structure without introducing systematic bias.

**Supplementary Table 2 VIF values for the included variables**

| **Variables** | **VIF** |
| --- | --- |
| Age, years | 1.0696 |
| Length of ICU stay, days | 6.5760 |
| NLR | 1,4028 |
| LYM , ×10⁹/L | 1.5037 |
| APTT, s | 1.0867 |
| Creatinine, μmol/L | 1.15432 |
| D-dimer | 1.1016 |
| CVC ues (yes/no) | 2.7932 |
| Length of CVC, d | 10.2151 |
| Length of tracheal intubation, d | 4.5334 |
| ventilator use **(yes/no)** | 8.2117 |
| Length of ventilator use, d | 3.59.6 |
| Tracheostomy**(yes/no)** | 2.4915 |
| Length of tracheotomy, d | 5.3261 |
| Pulmonary infection | 1.1429 |
| Gastrointestinal bleeding | 1.1889 |
| Electrolyte disturbance | 1.1637 |
| COUNT score | 1.5086 |
| Sedation | 3.6287 |
| Analgesia | 9.1787 |
| Antibiotic use | 1.2430 |
| Dehydrating agent use | 1.1548 |

Abbreviations: VIF, variance inflation factor.ICU, intensive care unit; NLR, neutrophil-to-lymphocyte ratio; LYM, lymphocyte count; APTT, activated partial thromboplastin time; CVC, central venous catheter; CONUT, Controlling Nutritional Status;
